# Thermally Induced Phase Transformation of Ni-Exchanged LTA Zeolite as an Alternative Route of Obtaining Stable Ni-Spinel Pigment

**DOI:** 10.3390/ma18143225

**Published:** 2025-07-08

**Authors:** Miomir Krsmanović, Aleksandar Popović, Smilja Marković, Bojana Milićević, Dušan Bučevac, Marjetka Savić, Mia Omerašević

**Affiliations:** 1 Vinča Institute of Nuclear Sciences, National Institute of the Republic of Serbia, University of Belgrade, 11000 Belgrade, Serbia; miomir.krsmanovic@vin.bg.ac.rs (M.K.); bucevac@vin.bg.ac.rs (D.B.); metk@vin.bg.ac.rs (M.S.); 2 Faculty of Chemistry, University of Belgrade, 11000 Belgrade, Serbia; apopovic@chem.bg.ac.rs; 3 Institute of Technical Sciences of SASA—Serbian Academy of Science and Arts, 11000 Belgrade, Serbia; smarkovic@itn.sanu.ac.rs; 4 Centre of Excellence for Photoconversion, Vinča Institute of Nuclear Sciences, National Institute of the Republic of Serbia, University of Belgrade, 11000 Belgrade, Serbia; bojana.milicevic85@gmail.com

**Keywords:** ion exchange, LTA zeolite, nickel immobilization, thermally induced phase transformation, nickel-spinel, blue-green pigments

## Abstract

This study investigates the thermally induced phase transformation of Ni-exchanged LTA zeolite as a dual-purpose method for nickel immobilization and the synthesis of stable ceramic pigments. The process describes a cost-effective and sustainable alternative to conventional pigment production, aligning with circular economy principles. Upon thermal treatment at temperatures ranging between 900 °C and 1300 °C, Ni-exchanged LTA zeolite undergoes a transformation to NiAl_2_O_4_ spinel, confirmed by XRPD, FTIR, and thermal analysis. Initially, NiO is formed, but as the temperature increases, it dissolves and transforms into NiAl_2_O_4_. Colorimetric studies revealed intensified blue pigmentation with increasing temperature, correlating with crystallite growth and structural evolution. SEM analysis showed morphological changes from cubic particles to sintered agglomerates, enhancing pigment stability and hardness. The Ni-LTA sample calcined at 1300 °C showed the highest hue angle, which was consistent with the formation of over 99 wt.% of the nickel aluminate crystalline phase at this temperature. The results demonstrate that Ni-LTA zeolite can be effectively transformed into durable greenish-blue pigments suitable for application in porcelain. This transformation is especially evident at 1300 °C, where a spinel phase (NiAlSi_2_O_4_) forms, with colorimetric values: L = 58.94, a* = –16.08, and b* = –15.90.

## 1. Introduction

Over the past decades, a high rate of industrialization was recorded, which resulted in the production and generation of large volumes of wastewater. Therefore, the amount of heavy metals disposed of into the environment through industrial wastewaters has increased and has been growing year by year [1]. Although nickel in small quantities plays an important role in metabolism as an enzyme activator, it is one of the most hazardous heavy metals. Water contamination with nickel is mainly generated from galvanization, mining, smelting, battery production, dyeing operations, alloying, and metal finishing [2]. The released metal cannot be eliminated naturally due to very slow absorption by plants. This is especially true when nickel is in chelate form [1]. Long and constant exposure to nickel can induce a range of different diseases, including dermatitis, headache, nausea, asthma, cardiovascular, and kidney diseases [3]. Bearing in mind that nickel is also carcinogenic [4], it is crucial to remove Ni from wastewater and prevent its entry into soil and groundwater. Various treatment processes for immobilization or separation of nickel ions, such as chemical precipitation [5], membrane dialysis [6], solvent extraction [7], flotation [8], nano and ultrafiltration [9,10], ion exchange [8], and adsorption [11,12], have been developed. One of the most effective methods for immobilization has been based on sorption processes such as adsorption as well as ion exchange. Various materials such as carbon materials [13], anion-exchange resins [14], modified metal oxides [15], clay materials [16], and fibers [17] have been investigated as sorbents. Compared to other nickel remediation methods and materials, as mentioned above, zeolite-based approaches offer several distinct advantages. While chemical precipitation is simple and cost-effective, it often results in the generation of large volumes of sludge that require further disposal. Membrane and filtration technologies, although efficient, are energy-intensive and prone to fouling. Solvent extraction involves the use of potentially hazardous organic solvents and is less environmentally friendly. Adsorbents like carbon materials and modified oxides demonstrate good performance, but their regeneration and reuse can be challenging. In addition to these materials, zeolite possesses unique ion exchange and sorption properties, which lead to a very high exchange capacity [18,19]. Both natural and synthetic zeolites possess a three-dimensional aluminosilicate cage structure with exchangeable cations such as Na and Ca, making them highly effective for the elimination of Ni from wastewater [20]. After ion-exchange, Ni ions are captured in the zeolite matrix.

In addition to having excellent sorption properties, zeolites can be successfully used as precursors for the synthesis of aluminosilicate-based ceramics [21,22]. The synthesis is based on the zeolite’s ability to transform into a new, stable structure (aluminosilicate ceramics) at high temperatures. The newly formed stable structure may include the ions captured in the zeolite matrix after ion exchange. High-temperature phase transformations in network structures, such as zeolites, enable the precise design of new phases with nanoscale control over stoichiometry and phase structure. This transformation, induced by heat, typically occurs through several stages [23,24,25] starting with a collapse of the structure and amorphization of materials, followed by the recrystallization into a new structure. The advantages of this manufacturing procedure over traditional methods of obtaining ceramics, such as firing of oxides or the sol–gel method, have been described [26,27]. It has been known that if transition metal cations are incorporated into the zeolite structure during ion exchange, the powder of ion-exchanged zeolite would be distinctively colored [28,29,30]. The coloration could be preserved even upon heating. This procedure ensures a uniform distribution of the extra-framework cations in zeolites, leading to an even spread of coloring centers within the structure, which enables more consistent and reliable coloring of the ceramic products after thermal treatment. The zeolites exchanged with transition elements such as Ni and Co normally transform into stable spinel-based ceramics [30,31,32] with the general formula AB_2_O_4_, where A^2+^ and B^3+^ are divalent and trivalent cations, respectively. It has been reported that the introduction of cobalt ions into the zeolite structure may result in the formation of zeolite-based blue pigment. The zeolite is usually exchanged with cobalt ions from water solutions of commercial cobalt chloride hexahydrate, CoCl_2_·6H_2_O, or cobalt nitrate hexahydrate, Co (NO_3_)_2_·6H_2_O [29,30]. Our preliminary research confirmed that thermal treatment of nickel-ion-exchanged zeolite can also lead to the formation of blue-green pigment.

The aim of this study was to investigate a thermally induced phase transformation of LTA (Linde-Type A) zeolite exchanged with Ni ions as a potential waste treatment method to accomplish efficient retention, specifically encapsulation of Ni ions. It will also be shown that this phase transformation is an alternative route of obtaining stable Ni-spinel (NiAl_2_O_4_), which can be used as an inorganic pigment following the rules of the circular economy. Pure and uniform commercial pigments can be synthesized starting from a mixture of commercial powders of NiO, Al_2_O_3,_ and SiO_2_ with the help of a synthesis process consisting of fine grinding, mixing, and heat treatment at high temperatures, usually 1400 °C.

Unlike this relatively expensive process, the method presented in this study is a unique and inexpensive route that comprises both the immobilization of Ni ions removed from the environment and the fabrication of stable, colored products.

## 2. Materials and Methods

### 2.1. Experimental Procedure

The Na Ca form of LTA (Si/Al = 1.00) zeolite manufactured by Union Carbide Co. (North Seadrift, TX, USA) was used as starting material. Zeolites are often processed into spherical forms using a clay binder; therefore, the zeolite powder was crushed and pulverized prior to use. An almost complete exchange to the Ni(II) form of Ca, Na-LTA type zeolite was achieved after three consecutive cation exchange cycles using a 0.1 M NiSO_4_ solution (NiSO_4_·7H_2_O, SIGMA Aldrich, St. Louis, MO, USA) with a solid-to-liquid ratio of 1:20. Ion exchange was carried out by stirring the zeolite sample in a 0.1 M aqueous solution of NiSO_4_ for 5 h at approximately 60 °C. This process was repeated 3 times. Following the ion exchange, the samples were thoroughly rinsed with distilled water to eliminate remaining sulfate ions and subsequently dried at 70 °C before further thermal treatment. The obtained samples of Ni-exchanged LTA zeolite were heat-treated for 2 h in an air atmosphere at temperatures ranging from 900 to 1300 °C. These temperatures are suitable for studying the products formed through phase transformations, which would behave as stable porcelain pigments.

### 2.2. Sample Characterization

To determine the elemental composition of the samples before and after ion exchange, XRF analysis (X-Ray Fluorescence Spectrometry) was performed using a Thermo Scientific ARL Perform’X Sequential X-ray fluorescence spectrometer (Waltham, MA, USA), equipped with a Rhodium tube, seven monochromators, and a wavelength-dispersive spectrometer. Powders of Na, Ca-LTA, and Ni-LTA were mixed with boric acid and mechanically pressed into pellets measuring 25 mm in diameter and 3.6 mm in height. The experiments were carried out under vacuum conditions to prevent interactions between X-rays and airborne particles. The data were obtained and processed using the Thermo Scientific UniQuant 3.0. Analysis Software. The elemental constitutions were determined based on the oxide weight fractions in the anhydrous zeolite form.

The phase composition of the samples was analyzed by X-ray powder diffraction (XRPD). XRPD patterns were obtained by RIGAKU Ultima IV diffractometer (Tokyo, Japan), with filtered Cu Kα radiation in the 2θ range from 4° to 80° using a step size of 0.02°. The present phases were identified, and the NiO/spinel ratio was estimated with the help of PDXL2 software (version 2.0.3.0), with reference to the patterns of the International Center for Diffraction Data (ICDD) version 2023. Detection limits for well-crystallized phases under optimized scan conditions are 1–3 wt.%. The Scherrer equation was applied to the X-ray diffraction data to calculate the average crystallite sizes for all peaks: *D* = *Kλ/βcosθ* where K is the shape factor, λ is the X-ray wavelength, *θ* denotes the diffraction angle, and *β* is the corrected full width at half maximum (FWHM) of the peaks. Calculations were also performed using PDXL2 software (version 2.0.3.0).

The amorphous/crystalline phase ratio in samples calcined at temperatures ranging from 900 to 1300 °C was obtained by the integration method with the help of the Origin program (2024 21-day free trial). This procedure utilizes a straight line as a baseline, established using the highest 2*θ* values to approximate the continuous background signal, and compares the area beneath the entire diffraction pattern with that beneath the crystalline peaks, according to the following equation [33,34]:

$$Crystallinity\;\left(\%\right)=100\times \frac{Area\;crystalline\;XRD\;peaks}{Area\;under\;all\;XRD\;peaks}$$

The thermal stability of Ni-LTA zeolite was investigated by simultaneous thermal gravimetry and differential thermal analysis (TG/DTA, SETSYS, SETARAM Instrumentation, Caluire-et-Cuire, France). Zeolite samples (approximately 10 mg) were analyzed under the air flow of 20 mL·min^−1^, heating with a rate of 10 °C min^−1^ from 30 to 1000 °C. The morphological characteristics of Ni-LTA zeolite, both before and after heat treatment, were analyzed using a scanning electron microscope (MIRA3 TESCAN from Brno, Czech Republic). Morphological analysis of Ni-LTA zeolite before and after the heat treatments was performed using a MIRA3 TESCAN (Brno, Czech Republic) scanning electron microscope (SEM). The particle size distribution was determined by a laser light-scattering particle size analyzer (Mastersizer 2000, Malvern Instruments Ltd., Malvern, Worcestershire, UK). Before measurement, the powders were dispersed in propan-2-ol using a vortex mixer for 10 s. The measurements were carried out in a Hydro µP unit filled with propan-2-ol, in a dynamic condition with a pump rotation of 1100 rpm.

Fourier Transform Infrared (FT-IR) spectroscopy analyses were carried out using a PerkinElmer Spectrum Two instrument to investigate Na, Ca, and Ni-exchanged LTA zeolite samples subjected to heat treatment at temperatures ranging from 900 °C to 1300 °C. Before analysis, the samples were prepared as potassium bromide (KBr) pellets (Fisher Chemical, Waltham, MA, USA). The FT-IR spectra were collected in the mid-infrared (MIR) region, covering the spectral range of 4000 to 400 cm^−1^.

A spectrophotometer, Shimadzu UV-2600 (Shimadzu Corporation, Tokyo, Japan), equipped with an integrated sphere (ISR-2600), was used for the diffuse reflection measurements in the 380–720 nm range with a 1 nm step size. Barium sulfate was used as a reference material. The colorimetric values (L*, a*, and b*) were calculated with the help of the CIELAB color space based on the measured diffuse reflection data and D65 standard illuminant (Standard Daylight) using Commission International de l’Eclairage (CIE 1931) standard colorimetric observer data. The quantifiers in the CIELAB color space are the lightness (L*, ranges of 0 to 100) and directions of the green-red coordinate (a*) and the blue-yellow coordinate (b*), as well as the amount of saturation (chroma, C). The chroma was calculated via $C=\sqrt{{a*}^{2}+{b*}^{2}}$. The h° parameter refers to the hue angle (tan^−1^ b*/a*), which describes the color’s position around a color wheel.

## 3. Results and Discussion

The extent of Na^+^ cation exchange was determined by evaluating the difference in sodium concentration within the zeolite before and after the ion exchange process. The chemical compositions of Na, Ca-LTA zeolite, determined by XRF technique, before and after exchange with Ni ions, are presented in Table 1. During the ion exchange process, Ni^2+^ ions moved through the pores and channels of the zeolite lattice and replaced exchangeable cations (mainly Na) from the zeolite. The obtained nickel form of the zeolite contains 26.78 wt.% of Ni ions. Loss on ignition was determined by the weight reduction observed during DTA/TG analysis conducted in air up to 1000 °C. The mass loss was 25% for Ni-LTA and 20% for Na, Ca-LTA.

The results of the XRF analysis were also used to estimate the extent of ion exchange, based on the differences in elemental composition before and after the process. The analysis focused on Na^+^, K^+^, and Ca^2+^, the primary cations available for exchange with Ni^2+^, enabling an estimation of the amount of Ni^2+^ incorporated into the material. A total of approximately 0.481 equivalents of cations (Na^+^, K^+^, Ca^2+^) were removed, and approximately 0.913 equivalents of Ni^2+^ were added, which is significantly more than the number of cations removed. These findings suggest that, in addition to ion exchange, Ni^2+^ retention occurred through additional mechanisms, most likely including physical adsorption on the surface and precipitation in the form of nickel hydroxide or oxide.

**Figure 1:**
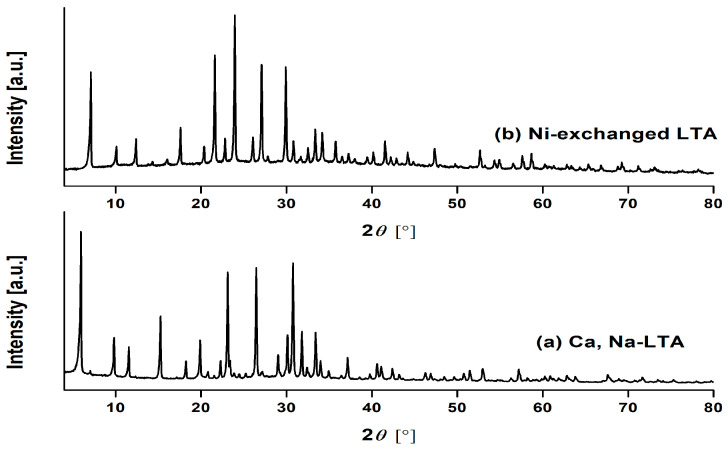
XRPD patterns of (**a**) Na, Ca-LTA zeolite; PDF card: 01-074-2534, and (**b**) Ni-exchanged LTA zeolite (Ni-LTA); PDF card: 00-057-0012.

XRPD patterns of Na, Ca-LTA zeolite before and after ion exchange with Ni ions are presented in Figure 1. The substitution of Na^+^, Ca^2+^, as well as trace K^+^ and Fe^3+^ ions with Ni^2+^ within the zeolite framework leads to the production of Ni-LTA zeolite [35]. The successive ion exchange procedure caused a broadening of the LTA zeolite diffraction lines. The crystallinity is noticeably reduced, indicating that the introduction of Ni^2+^ ions induced notable alterations in the structure of the zeolite. Some characteristic peaks of starting Na, Ca-LTA completely disappeared (Figure 1a), whereas a new, stable crystalline phase Ni-LTA was formed (PDF card: 00-057-0012). Therefore, it can be concluded that ion exchange was successful.

**Figure 2:**
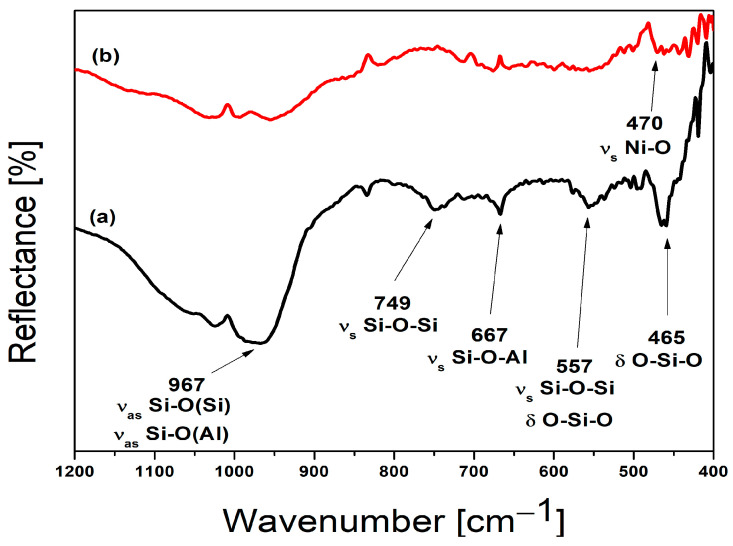
FTIR spectra of (**a**) Na, Ca-LTA zeolite and (**b**) Ni-exchanged zeolite LTA (Ni-LTA).

The mid-FTIR spectra (1200–400 cm^−1^) of Na, Ca-LTA zeolite (a) and nickel-exchanged zeolite LTA (b) are given in Figure 2, as well as Table 2. A large, broad band observed in Na, Ca-LTA spectrum at a wavenumber of 967 cm^−1^, can be attributed to the overlap of the asymmetric vibrations of Si–O (bridging) and Si–O- (nonbridging) bonds. In the Ni-LTA spectrum, this band is significantly reduced and shifted towards higher wavenumbers. As can be seen from Figure 2, characteristic bands for LTA zeolite at 749 cm^−1^ and 667 cm^−1^ completely disappear after ion exchange with Ni cations. These two bands can be assigned to symmetric stretching vibrations of bridge bonds Si-O-Si and Si-O-Al, respectively [36]. The prominent bands observed at 557 cm^−1^ and 465 cm^−1^ in the starting Na-LTA zeolite are reduced in intensity in the Ni-LTA form, indicating a change in the symmetry of the D4R unit of the zeolite [37]. Consequently, ion exchange led to variations in both the position and intensity of the bands. These differences observed between the two spectra can be attributed to the distinct ionic radii and atomic masses of the exchanged cations [35]. FTIR results are in good agreement with results obtained by XRPD analysis. FTIR spectrum of Ni-LTA possesses new bands (471, 443, and 409 cm^−1^) which correspond to Ni-O and Ni-O-Al stretching vibrations [36,38].

**Figure 3:**
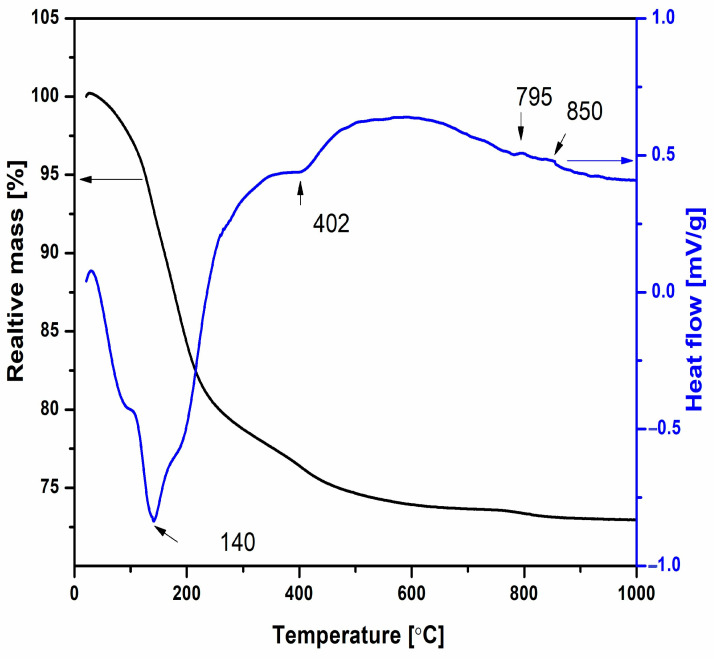
TG and DTA curves of Ni-exchanged LTA zeolite (Ni-LTA) heating with a rate of 10 °C min^−1^ in the air.

The thermal behavior and weight changes in Ni-LTA powder upon heating up to 1000 °C were analyzed using thermogravimetric/differential thermal analysis (TG/DTA), as shown in Figure 3. An initial weight loss of approximately 18%, observed in the TG curve between 30 and 300 °C, is attributed to the desorption of physically adsorbed water. This endothermic process is further supported by a pronounced endothermic peak in the DTA curve, with a minimum at 140 °C. A subsequent weight loss of about 6%, occurring between 300 and 700 °C, corresponds to the release of structural water and is accompanied by an endothermic minimum at 402 °C in the DTA curve. Minor DTA fluctuations observed above 720 °C are associated with an additional ~1% weight loss, which is likewise attributed to the continued release of structural water. The observed mass losses in both the low-temperature range (30–300 °C) and the intermediate range (300–700 °C) are consistent with the release of physically adsorbed and structural water, respectively. These processes are typically irreversible, particularly in the case of structural water, as its removal is associated with partial decomposition or structural rearrangement of the zeolite framework. The minor weight loss above 720 °C suggests the continued gradual release of tightly bound water or possible framework dehydroxylation, but with no evidence of rehydration or reversible behavior upon cooling in this thermal regime. Moreover, the TG curve does not show any mass gain at higher temperatures.

The XRPD analysis previously obtained by Dondur et al. [31] suggests that these deviations may be attributed to the structural changes, such as the transformation of zeolite structure into amorphous and/or crystalline phase, which takes place above 780 °C. As can be seen from Figure 3, the amorphization of the zeolite and the crystallization of new phases are gradual processes. DTA/TG analysis was performed to estimate the temperature at which the amorphous structure transforms into the new crystalline phase. Knowledge of the crystallization temperature is essential for this research.

**Figure 4:**
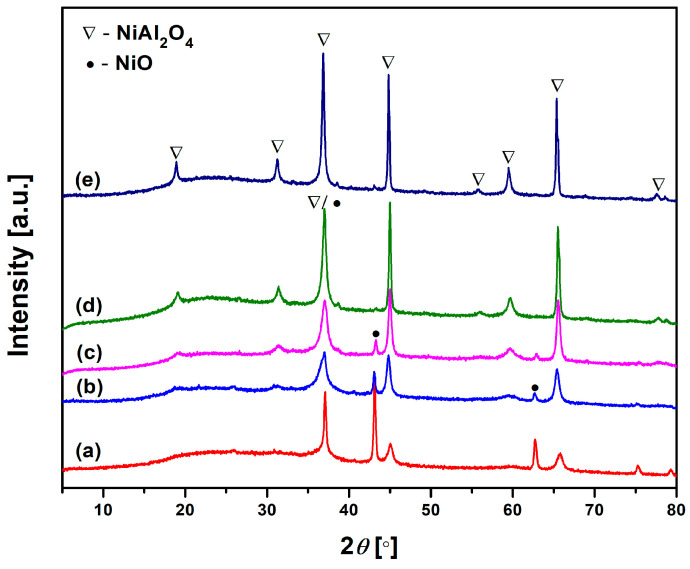
XRPD patterns of Ni-LTA thermally treated at (**a**) 900 °C, *(***b**) 1000 °C, (**c**) 1100 °C, (**d**) 1200 °C, and (**e**) 1300 °C for 2 h.

Therefore, to examine the new crystalline phase resulting from the thermal transformation of Ni-LTA zeolite, the precursor Ni-LTA was heated in a furnace at temperatures ranging from 900 to 1300 °C, in 100 °C increments. XRPD analysis was used to analyze phase transformations during calcination. As can be seen from Figure 4, the thermal treatment above 900 °C induced recrystallization of Ni^2+^ exchanged zeolite LTA into NiO, Ni-spinel (NiAl_2_O_4_), and amorphous silicate. An illustrative chemical reaction of the zeolite phase transformation during heating can be represented by the following chemical equation: 4NiAlSiO_4_ + O_2_ → 2NiAl_2_O_4_ + 2NiO + 4SiO_2_. None of the SiO_2_ crystal phases were detected in the whole temperature range up to 1300 °C (Figure 4). The formation of an amorphous silicate phase is expected, especially as a by-product of zeolite framework collapse at elevated temperatures. In such conditions, silica cannot easily recrystallize without the presence of specific mineralizers or nucleating agents [39], as the transformation from amorphous to crystalline SiO_2_ is kinetically hindered in pure systems [40,41].

The fractions of amorphous and crystalline phases in the samples heated at temperatures ranging from 900 to 1300 °C were determined and shown in Table 3. As expected, the fraction of the amorphous phase decreased with increasing temperature while the fraction of the crystalline phase increased. The sample heat-treated at 900 °C consisted of ~65 wt.% of amorphous and ~35 wt.% of crystalline phase. As the calcination temperature increased, the fraction of crystalline phases increased. As can be seen, even at 1300 °C, the crystalline phase does not exceed 45 wt.%, which means that the material remains predominantly amorphous. This high stability of the amorphous phase may be desirable, especially for certain pigment applications, where it can contribute to color intensity and transparency. However, for thermal and chemical stability, the crystalline phase is preferred as it is thermodynamically more stable.

As confirmed by Figure 4, the crystalline phase of heat-treated samples consists of NiO and NiAl_2_O_4_, regardless of heat-treatment temperature. The fractions of these compounds in the crystalline phase of samples heat-treated at different temperatures (900–1300 °C) were determined and listed in Table 4. The fraction values are also accompanied by the crystallite size of both phases.

As can be seen, the content of NiO decreased, while the content of NiAl_2_O_4_ spinel increased with heat-treatment temperature. The crystalline phase of the sample heat-treated at 900 °C consisted of ~84 wt.% of NiO and 16 wt.% of NiAl_2_O_4_. Further increase in heat-treatment temperature promoted ordering of Ni-spinel crystal structure, resulting in an increase in NiAl_2_O_4_ spinel fraction and a decrease in a NiO fraction. The fraction of NiO in the crystal phase of the sample heat-treated at 1300 °C was only 0.84 wt.%, whereas the fraction of NiAl_2_O_4_ spinel reached a value of over 99 wt.%. It is important to stress that NiO crystallizes prior to the spinel formation. This can be explained by the fact that [32] crystal field stabilization energy drives Ni^2+^ to adopt octahedral coordination instead of a tetrahedral one, which is typical for nickel cations in dehydrated LTA zeolite. The octahedral coordination of nickel cations in nickel oxide is thermodynamically preferred, facilitating the formation of NiO. Consequently, the crystallite size of the NiO phase in the sample heat-treated at 900 °C attained the highest value of 156 Å. A further increase in temperature promotes the formation of NiAl_2_O_4_ spinel (Figure 4), which is followed by a decrease in NiO crystallite size and an increase in crystallite size of the newly created spinel (Table 4). Larger crystallites and inter-particle necking contribute to a reduction in surface area and an increase in particle cohesion, which in turn decreases their wettability and dispersibility in liquid media. An ideal pigment must strike a balance between: crystalline phase stability (NiAl_2_O_4_ spinel, known for its thermal and chemical stability) and particle characteristics that favor dispersibility (fine particle size and minimal sintering). Intermediate temperatures (~1100–1200 °C) may represent an optimal range for pigment synthesis, as they allow for adequate formation of NiAl_2_O_4_ spinel, ensuring good stability, and prevention of excessive sintering and crystallite growth, which would otherwise compromise potential dispersibility.

**Figure 5:**
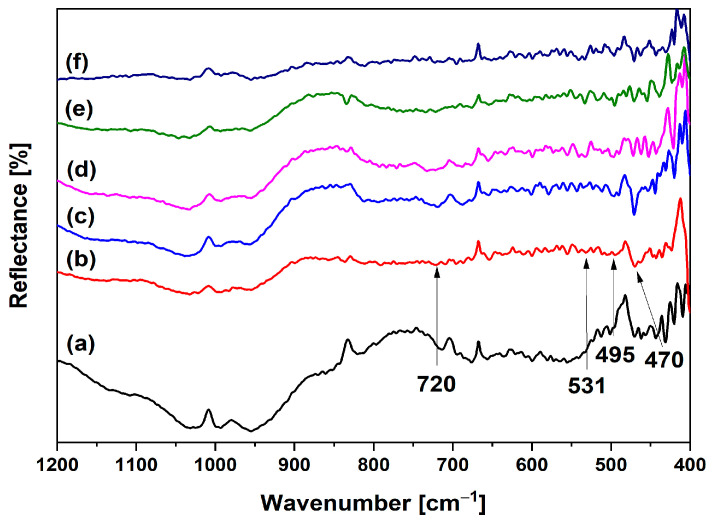
FTIR spectra of Ni exchanged samples-heat treated at: (**a**) room temperature; (**b**) 900 °C; (**c**) 1000 °C; (**d**) 1100 °C; (**e**) 1200 °C, and (**f**) 1300 °C.

To gain deeper insight into the structural changes occurring during ion exchange and thermal treatment, FT-IR analysis was conducted on heat-treated samples. The FT-IR spectra of Ni-LTA zeolite at room temperature and after heating at 900–1300 °C are presented in Figure 5.

As can be seen from the FTIR spectrum (Figure 5), a characteristic band of Ni-LTA at 967 cm^−1^ completely disappears during heating. This is one more confirmation of the transformation of Ni-LTA into Ni-spinel. The bands at 1030 cm^−1^ and 813 cm^−1^ are assigned to the asymmetric and symmetric vibrations of Si-O-Si bonds [42]. The infrared (IR) spectral pattern characteristic of spinel-like structures represents two Al–O stretching bands within the 500–900 cm^−1^ range, which can be attributed to distinct coordination environments of aluminum atoms (AlO_6_ and AlO_4_ units). This observation is attributable to the overlap of transition metal–oxygen vibrational modes with the Al–O bands in the spectrum [32,43,44]. Bands around 720, 531, 495, and 470 cm^−1^ correspond to the regular spinel structure with only sixfold coordinated aluminum [18,45,46], which would confirm the formation of NiAl_2_O_4_ spinel structure. This is in a good agreement with the XRD results presented in Figure 4. These absorption bands correspond to the symmetric and asymmetric stretching, as well as bending vibrations of Ni–O, Al–O, and Ni–O–Al bonds located at tetrahedral and octahedral sites within the metal aluminate spinel structure [35,47,48]. The sample at 900 °C possesses the highest amount of NiO. Therefore, the band at 470 cm^−1^ (corresponding to Ni-O stretching vibrations) is the most pronounced at this temperature [38,49].

### 3.1. Colorimetric Analysis

**Figure 6:**
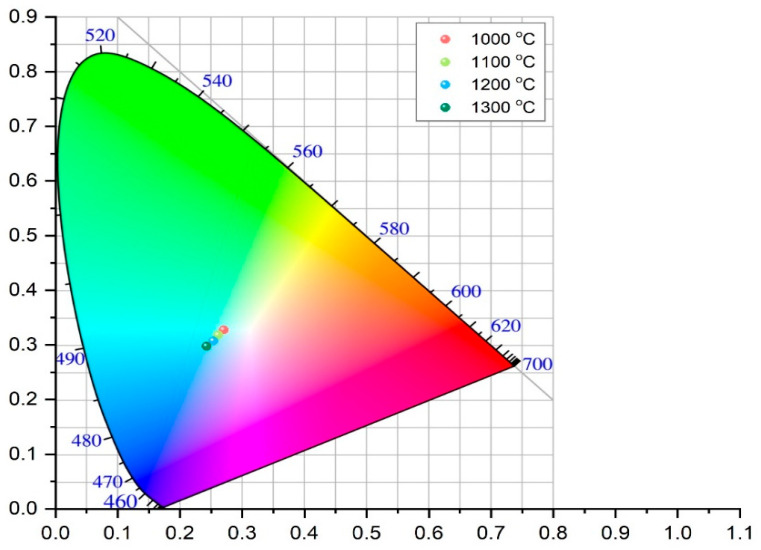
Presents colorimetric outcomes: CIE 1931 xy diagram for all samples.

Previous analyses have demonstrated the formation of the spinel structure at 1000 °C; therefore, colorimetric measurements were conducted exclusively on samples heat-treated at temperatures between 1000 °C and 1300 °C. The colorimetric results for these samples are summarized in Table 5, while Figure 6 presents the CIE 1931 chromaticity diagram (x, y), pinpointing the exact color coordinates of each sample. As Table 5 shows, increasing calcination temperature leads to a decline in a* values, indicating a reduction in green intensity, alongside more negative b* values, reflecting a shift toward blue. Concurrently, L* decreases with rising temperature. These trends are clearly visible in Figure 6, where the diminishing (more negative) a* values correspond to a loss of green, and increasingly negative b* values show enhanced blueness.

Comparison of the results given in Table 5 reveals that the increase in b* value in the negative direction is more pronounced than the decrease in a* value in the negative direction, which confirms the presence of two mixed colors, green and blue, where the blue color exhibits higher intensity. The decrease in the L* parameter corresponds to the reduced lightness of the sample. All these findings are in good agreement with XRPD and FTIR results presented in Figure 4 and Figure 5, respectively. These gradual changes in the colorimetric results, i.e., color shift, are a direct result of the incremental transformation of the crystal structure during calcination, which was followed by the decrease in NiO content and increase in the NiAl_2_O_4_ spinel content.

**Figure 7:**
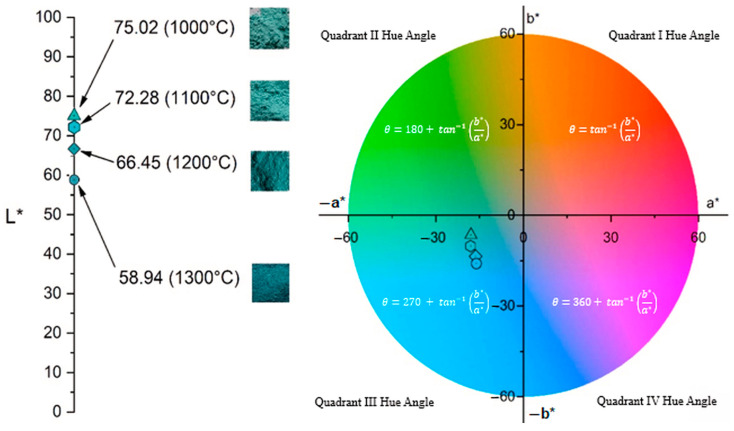
Color wheel of Ni-LTA powder samples treated at different temperatures and corresponding photographs for visual comparison.

In addition to the L*, a*, and b* values, Table 5 also reports the C* (chroma) and h° (hue angle) parameters for each sample. The C* value, derived from the a* and b* components, indicates the saturation or vividness of the color—higher C* values correspond to more intense and saturated hues, as reflected in the rendered colors shown in Table 5. In contrast, the h° value represents the hue angle, which defines the color’s position on the color wheel, as illustrated in Figure 7. The h° values on the color wheel indicate the pure hue of the samples and their tonal characteristics [50]. As can be seen from Figure 7, h° values of all samples are in a green/blue (−a/−b) quadrant and increased with increasing temperature of calcination. The Ni-LTA sample heated at 1300 °C exhibited the highest h° value, which is expected due to the formation of over 99 wt.% of the crystalline nickel aluminate phase at this temperature. According to the ISCC–NBS color system, this corresponds to a strong greenish-blue hue.

### 3.2. Color Characterization of Ceramic Pigments

**Figure 8:**
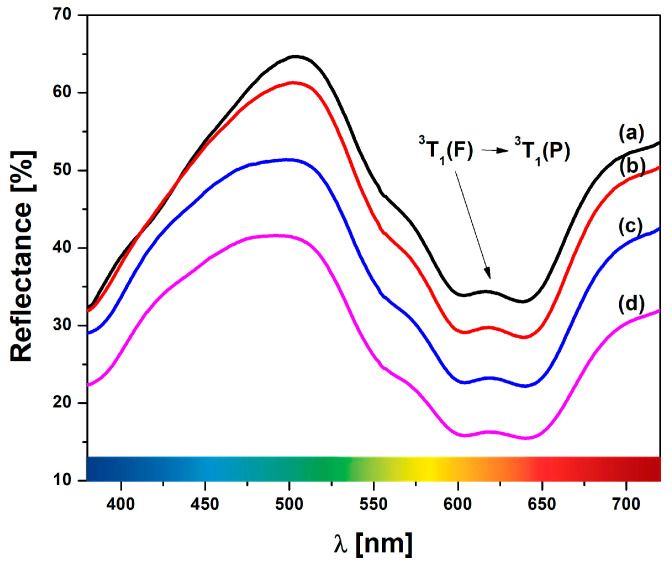
UV-Vis diffuse reflectance spectra of Ni-LTA thermally treated at different temperatures: (**a**) 1000 °C; (**b**) 1100 °C; (**c**) 1200 °C, and (**d**) 1300 °C.

Diffuse reflectance spectra for Ni-LTA heated at 1000 °C, 1100 °C, 1200 °C, and 1300 °C are given in Figure 8. Notably, a distinct band within the range of 490–505 nm is observed, corresponding to the presence of Ni ions within the structure. The sample heated at 1000 °C spectrum shows the appearance of characteristic bands around 505 and 617 nm. These bands correspond to the pale green-blue (cyan) color of the sample under investigation. As the calcination temperature increases, the band at 505 nm shifts to the cyan band until it reaches ~490 nm. The small band, which corresponds to the complementary peaks at 617 nm, shifts to 618, 619, and 620 nm. This band could be attributed to the ^3^T1 (F)/^3^T1 (P) transitions, which correspond to tetrahedral nickel in the spinel phase, verified by the XRPD results [51].

### 3.3. SEM Analysis

**Figure 9:**
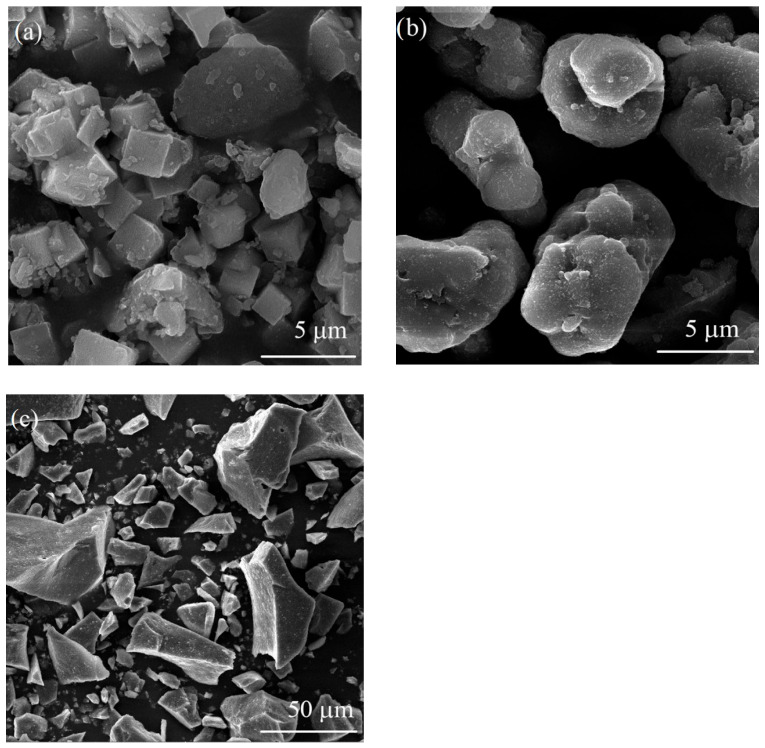
SEM micrographs of Ni-LTA (**a**) at room temperature, (**b**) at 1000 °C, and (**c**) at 1300 °C.

Figure 9 presents SEM micrographs of the Ni-exchanged LTA powders, untreated and after thermal treatment at 1000 °C and 1300 °C. The powders were manually crushed in an agate mortar with a pestle before SEM analysis. Figure 9a shows uniform particle morphology of Ni-exchanged LTA powder, mainly consisting of cubic-shaped particles few microns in size. The presence of agglomerates having a diameter less than 8 µm was also observed. As Figure 9b evidences, the shape and size of particles were affected to a large extent by the thermal treatment at 1000 °C. Coalescence of small, cubic-shaped particles resulted in the formation of larger, mainly rounded particles between 5 and 15 µm in size. Further increase in heat treatment temperature to 1300 °C initiated sintering of zeolite particles and consequent formation of hard, faceted particles as shown in Figure 9c. The particles were from 5 to 60 µm in size. For this reason, the sample thermally treated at 1300 °C was analysed under lower magnification in order to capture a larger number of particles. It is important to mention that the pigment performance is mainly affected by the phase composition, which is in turn affected by the temperature of thermal treatment. Therefore, it is difficult to distinguish the effect of particle morphology from the effect of phase composition, as both of them change with temperature. In general, the effect of powder pigment morphology is considered to be minor, as the thermally treated powders are normally subjected to subsequent milling in order to obtain fine pigment powders ready for ceramics dyeing. The powders thermally treated at different temperatures were milled for 3 h in a ball mill using Al_2_O_3_ balls as a grinding medium. The particle size distribution of these powders was examined in the following section.

### 3.4. Particle Size Distribution

**Figure 10:**
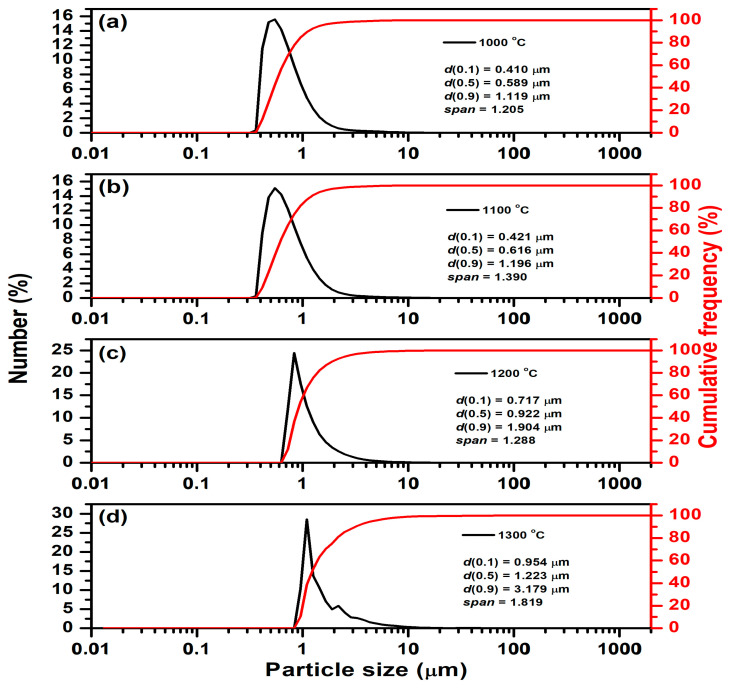
Particle size distribution of Ni-LTA zeolite powders heated at (**a**) 1000 °C, (**b**) 1100 °C, (**c**) 1200 °C, and (**d**) 1300 °C.

The results of particle size distribution measurement for samples thermally treated at different temperatures are listed in Table 6, as well as Figure 10. As expected, the measured particle size is smaller than that estimated from SEM micrographs due to additional milling. As can be seen, all presented parameters increase with the temperature of thermal treatment. The average particle size (d (0.5)) significantly increased from 0.589 µm in samples thermally treated at 1000 °C to 1.223 µm in samples thermally treated at 1300 °C. At the same time, the span increases from 1.205 (at 1000 °C) to 1.819 (at 1300 °C). This indicates that particle size variation becomes more pronounced at higher temperatures, suggesting both growth and uneven agglomeration of particles. Particle growth is temperature-dependent and is likely driven by sintering, coalescence, or grain growth mechanisms that are enhanced at higher temperatures, which is expected and consistent with the SEM analysis (Figure 9).

## 4. Conclusions

Ca, Na LTA zeolite showed good selectivity for nickel ions, which makes it a suitable material for immobilization of nickel from aqueous solutions. Following the ion exchange process, the nickel-exchanged LTA zeolite containing over 26 wt.% of Ni ions was successfully obtained. It was found that the phase transformation during thermal treatment of Ni-LTA between 900 °C and 1300 °C led to the gradual crystallization of NiO and NiAl_2_O_4_ phases. XRPD analysis revealed that NiO crystallizes initially and then dissolves as NiAl_2_O_4_ spinel crystallites with increasing calcination temperature. The FTIR spectra supported this transformation, showing the disappearance of Ni-LTA bands and the emergence of spinel-related vibrational bands. Moreover, the thermal analysis revealed that the structural water loss and phase transformations took place between 300 °C and 700 °C, with crystallization processes beginning at 900 °C. The colorimetric studies showed that the increase in calcination temperature progressively enhanced the blue color intensity in the pigments as a result of the structural transformation. The fraction of crystalline phases and the crystallite size of NiAl_2_O_4_ spinel increased with calcination temperature, resulting in a more stable color. The obtained results confirmed that Ni-exchanged LTA zeolite can be effectively converted into stable NiAl_2_O_4_ ceramic pigments, particularly exhibiting blue tones, which could be suitable for industrial applications as porcelain pigments. The low crystallization even at 1300 °C indicates the necessity for further optimization if a stable crystalline pigment is desired. However, if the color remains stable and intense despite a high amorphous content, the composition may be acceptable. Nevertheless, it is recommended to evaluate its long-term chemical and thermal stability. Future research will focus on the incorporation of mineral additives (nucleating agents) to promote crystallization.

## Figures and Tables

**Table 1:** Elemental composition of Na, Ca-LTA zeolite before and after ion exchange with 0.1 M solution of NiSO_4_.

Elements	Na	K	Ca	Fe	Al	Si	O	Ni
Before exchange (wt.%)	12.07	0.21	1.46	0.70	16.83	22.82	45.91	/
After exchange (wt.%)	2.08	0.051	0.61	0.001	14.283	19.79	36.41	26.79

**Table 5:** The colorimetric results for samples heat-treated at different temperatures, including L* (lightness), a* and b* values, C* (chroma), h° (hue angle), and * the rendered colors (obtained by Nixsensor color-converter by colorimetric coordinates).

Temperature [°C]	L*	a*	b*	C*	h°	Rendered Color *
1000	75.02	−18.07	−6.41	19.07	199.64	
1100	72.28	−17.96	−10.11	20.71	209.23	
1200	66.45	−16.35	−13.53	21.22	219.61	
1300	58.94	−16.08	−15.90	22.61	224.68	

**Table 6:** Parameters generated from a particle size distributeon analysis (d(0.1)—10% of particles smaller than this size, d(0.5)—50% of particles are smaller than this size, d(0.9)—90% of particles are smaller than this size. Span: measures the width of the distribution).

Temperature [°C]	d(0.1) [µm]	d(0.5) [µm]	d(0.9) [µm]	Span
1000	0.410	0.589	1.119	1.205
1100	0.421	0.616	1.196	1.390
1200	0.717	0.922	1.904	1.288
1300	0.954	1.223	3.179	1.819

**Table 2:** Wavenumbers of major FTIR bands of Na, Ca-LTA, and Ni-LTA zeolite.

Wavenumber [cm^−1^]	Vibration Type	Assignment
967	asymmetric stretching vibrations of bridge bonds	ν_as_ Si–O(Si) v_as_ Si–O-(Al)
749	symmetric stretching vibrations of bridge bonds	ν_s_ Si–O-Si
667	symmetric stretching vibrations of bridge bonds	ν_s_ Si–O-Al
557	symmetric stretching vibrations of bridge bonds and bending vibrations	ν_s_ Si–O-Si δ O-Si-O
465	bending vibrations (antiphase)	δ O-Si-O
470	symmetric stretching vibrations	ν_s_ Ni-O

**Table 3:** Content of amorphous and crystalline phases after a two-hour-long heat-treatment of Ni-LTA at different temperatures.

Temperature [°C]	Crystalline Phase [wt.%]	Amorphous Phase [wt.%]
900	35.27	64.73
1000	40.88	59.12
1100	41.42	58.58
1200	43.24	56.76
1300	45.39	54.61

**Table 4:** Percentage and crystallite size of NiO and NiAl_2_O_4_ during thermal treatment at temperatures from 900–1300 °C.

Temperature [°C]	NiO [%wt]	Crystallite Size of NiO [Å]	NiAl_2_O_4_ [%wt]	Crystallite Size of NiAl_2_O_4_ [Å]
900	83.80	156	16.20	/
1000	6.36	48	93.64	23
1100	3.15	31	96.85	43
1200	0.93	12	99.07	110
1300	0.84	/	99.16	141

## Data Availability

The original contributions presented in this study are included in the article. Further inquiries can be directed to the corresponding author.
